# Domestic Summary

**Published:** 1840-05-02

**Authors:** 


### DOMESTIC SUMMARY.

*On the power of Saccharine substances in general, and uncrystallizable sugar in particular, of protecting the solution of Protiodide of Iron from decomposition.*—By William Procter, Jr.—To be able to protect the solution of protiodide of iron from decomposition, and, consequently, to preserve its medicinal power unimpaired, has been a desideratum to the medicinal practitioner, as owing to the gradual but certain process of reduction of the strength of the solution, this preparation is one to which the physician is compelled to look with suspicion, not on account of any original want of power in the remedy, but by reason of the process of deterioration which is constantly progressing, often, notwithstanding the recommended precaution of keeping metallic iron in the solution.—It is true that, when this is done in a proper manner, there is a mere transfer of iron from the protecting metal to the iodine of the decomposed salt, as it is gradually effected, while its base is deposited in the state of peroxide. But even allowing this protecting power to be fully exercised, the ferruginous deposit, making the solution turbid, not to speak of the iron filings or wire, renders it inelegant, and frequently subjects the apothecary to the inconvenience of filtering the solution before dispensing it.

Thus far we have been viewing the subject within the precincts of the apothecary's store, but the most serious difficulties yet remain to be considered, viz. : after the remedy is placed in the hands of the patient. Some pharmaceutists take the precaution to introduce small quantities of metallic iron into the vials of the solution before selling it, but this is by no means general. As the remedy is taken in small doses, and its exhibition sometimes continued through considerable periods of time, the frequent opening of the vial, not to say the liability of leaving it unstopped, as must often be the case, causes the decomposition to go on rapidly when it is not in contact with iron. In corroboration of these remarks, I have the testimony of one of our first physicians, who informed me that he had discontinued the use of this preparation in his practice, in consequence of its great variation in strength.

I have deemed it proper to make the foregoing remarks as introductory to the following observations and experiments, which were made with the object of discovering a remedy for the evils which we have seen detailed.

The agents employed belong to the saccharine substances. The first application of one of this class of substances as a protective agent in pharmaceutical manipulation, was by Vallet, in his now celebrated ferruginous pills.

Shortly after the formula for his preparation was published in this country, I gave a process for preparing a tincture of *protomuriate of iron*, (Vol. X. p. 272, of this Journal,) which was kept in the state of a protosalt, through the intervention of honey. It was this idea which suggested the power of the same agent in preserving the solution of protiodide of iron, and the sequel will show how far the suggestion has been realized.

As it is our duty at the time we are pursuing a course of investigation, to note *all* the phenomena that offer, perhaps it will be well to give an idea of the *relative* protecting power of several saccharine substances, so as to be able to appreciate their importance, and to ascertain, if possible, some general principle which will account for the variation in their protective power.

The substances tried were sugar of milk, manna, cane sugar, honey, and uncrystallizable sugar.

The uncrystallizable portion of honey and molasses was obtained by mixing the honey or molasses with twice its weight of alcohol. The cane sugar precipitates, if molasses is employed, and the crystallizable honey, if honey is used, and by evaporating the solution, the preparation is obtained free from alcohol. They should be decolorized as much as possible, by boiling with animal charcoal, before being used.

1st. The power of sugar of milk and manna in protecting the iodide is very slight, although they each may possess the property in a limited degree. The trials which were made, however, offer convincing proof of their inadequacy to perform the purpose in view.

2d. Three drachms of the solution of protiodide of iron was mixed with one drachm of simple syrup, and placed in a vial exposed to air and light. In a similar vial a like quantity of the solution was placed without the addition of syrup, and equally exposed to air and light. At the end of forty-eight hours the saccharine solution remained transparent, possessing its original colour, while the other had acquired a brownish hue. They remained thus exposed to air and light for two weeks—at the end of that period the unprotected solution had deposited a considerable quantity of peroxide of iron, and was strongly charged with free iodine. The saccharine solution was also coloured, but in a very slight degree, with an equally small deposit of ferruginous oxide, but we must be aware that this occurred after a full exposure to *air and light* for two weeks.

3d. Three drachms of the solution, as before, was mixed with two drachms of simple syrup, and the mixture exposed for five days to air and light. On examination, the solution remained perfectly unaltered, not communicating the slightest tinge to starch water, or exhibiting any precipitate whatever.

The vial was then corked, and left exposed to the light. Twenty-two days after the commencement of the experiment, the first evidence of free iodine was manifested, and to this time, nearly two months from that date, the solution remains so little altered that the presence of free iodine is hardly perceptible, and the deposit of oxide equally minute; there being none whatever on the sides of the bottle.

4th. Three drachms of solution of protiodide of iron was mixed with one drachm of honey, and the mixture filtered, to render it perfectly transparent, and then exposed to air and light for three days without being the least affected. Twenty days after, the solution remained unchanged, and one month had elapsed before the slightest trace of free iodine could be detected. Two months after decomposition had progressed so tardily, that if no other protective agent existed, this would be better than iron.

5th. Three drachms of the iodous solution were mixed with one drachm of uncrystallizable honey. The mixture was treated precisely as in the last experiment, and was found to answer more effectually than the unaltered honey.

6th. Three drachms of the solution, as before, was mixed with two drachms of uncrystallizable sugar, (of molasses.) The mixture was then exposed several days to light and air without the slightest alteration. Nearly two months have elapsed since the beginning of the experiment, and not the slightest trace of free iodine, or of oxide, have been separated, notwithstanding it has been tested twenty times with solution of starch.

7th. To exhibit the protecting power of these agents more pointedly, two vials, with wide mouths were nearly filled with filtered starch water; to one was added a few drops of the protected solution, to the other an equal quantity of the unprotected solution of iodide of iron. At the end of twenty-four hours the presence of free iodine was rendered evident in the latter by the blue colour acquired by the starchy solution, whereas the former remained colourless.

8th. Thinking, notwithstanding the present protective power of the agents, that the increased temperature of summer might cause fermentation, and thus render the protector worse than useless, four ounces of the solution, protected with uncrystallizable honey, was placed in a vessel of water, the temperature of which varied from 80° to 100° Fah., for nine days. At the end of this period no signs of fermentation were evinced, and no free iodine existed in the solution. The exposure to heat was then discontinued, under the impression that if disposed to ferment, the solution had ample time to give notice of it.

It remains now to offer a formula for the proposed preparation. The strength of the following is that proposed to be adopted at the late convention on the Pharmacopœia:

℞.—Iodine, - - - ʒxi.
Iron filings - - - ʒiv.
Syrup, - - -
Uncrystallizable honey, or
Uncrystallizable sugar, - ℥iv.
Distilled water, a sufficient quantity.

Mix the iodine with eight fluid ounces of the distilled water, and gradually add the iron filings, stirring constantly; then apply a gentle heat until the solution shall have acquired a light green colour, or shall not give a blue colour to the solution of starch, then add whichever of the three protecting substances may be chosen, and continue the heat a short time, and filter. Lastly, wash the filter with as much distilled water as will make sixteen fluid ounces of solution of protiodide of iron.

When either sugar or honey is used, the colour of the solution is very little altered, while it is rendered much more palatable.

To the physician, the foregoing remarks are believed to be fraught with advantage, as it gives him the power to control the efficiency of his remedy by merely directing a quantity of simple syrup to be mixed with the solution, when he has reason to believe that it is not done previously, and increasing the dose proportionably. To the pharmaceutist it offers to be equally beneficial, by rendering a preparation, hitherto uncertain and inelegant, permanent in its medicinal power, and free from a sedimental deposit, which, he will admit, adds nothing to the appearance of his bottle.

*American Journal of Pharmacy.*

